# A Prospective, Multicenter, Single-Blind Study Assessing Indices of SNAP II Versus BIS VISTA on Surgical Patients Undergoing General Anesthesia

**DOI:** 10.2196/resprot.6741

**Published:** 2017-02-03

**Authors:** Sergio D Bergese, Alberto A Uribe, Erika G Puente, R-Jay L Marcus, Randall J Krohn, Steven Docsa, Roy G Soto, Keith A Candiotti

**Affiliations:** ^1^ Department of Anesthesiology The Ohio State University Wexner Medical Center Columbus, OH United States; ^2^ Department of Neurological Surgery The Ohio State University Wexner Medical Center Columbus, OH United States; ^3^ Department of Anesthesiology Northwestern University Prentice Women's Hospital Chicago, IL United States; ^4^ Stryker Corporation Kalamazoo, MI United States; ^5^ Department of Anesthesiology William Beaumont Hospital Royal Oak, MI United States; ^6^ Department of Anesthesiology University of Miami/Jackson Memorial Hospital Miami, FL United States

**Keywords:** Bispectral index, SNAP II, BIS VISTA, EEG monitoring

## Abstract

**Background:**

Traditionally, anesthesiologists have relied on nonspecific subjective and objective physical signs to assess patients’ comfort level and depth of anesthesia. Commercial development of electrical monitors, which use low- and high-frequency electroencephalogram (EEG) signals, have been developed to enhance the assessment of patients’ level of consciousness. Multiple studies have shown that monitoring patients’ consciousness levels can help in reducing drug consumption, anesthesia-related adverse events, and recovery time. This clinical study will provide information by simultaneously comparing the performance of the SNAP II (a single-channel EEG device) and the bispectral index (BIS) VISTA (a dual-channel EEG device) by assessing their efficacy in monitoring different anesthetic states in patients undergoing general anesthesia.

**Objective:**

The primary objective of this study is to establish the range of index values for the SNAP II corresponding to each anesthetic state (preinduction, loss of response, maintenance, first purposeful response, and extubation). The secondary objectives will assess the range of index values for BIS VISTA corresponding to each anesthetic state compared to published BIS VISTA range information, and estimate the area under the curve, sensitivity, and specificity for both devices.

**Methods:**

This is a multicenter, prospective, double-arm, parallel assignment, single-blind study involving patients undergoing elective surgery that requires general anesthesia. The study will include 40 patients and will be conducted at the following sites: The Ohio State University Medical Center (Columbus, OH); Northwestern University Prentice Women's Hospital (Chicago, IL); and University of Miami Jackson Memorial Hospital (Miami, FL). The study will assess the predictive value of SNAP II versus BIS VISTA indices at various anesthetic states in patients undergoing general anesthesia (preinduction, loss of response, maintenance, first purposeful response, and extubation). The SNAP II and BIS VISTA electrode arrays will be placed on the patient’s forehead on opposite sides. The hemisphere location for both devices’ electrodes will be equally alternated among the patient population. The index values for both devices will be recorded and correlated with the scorings received by performing the Modified Observer’s Assessment of Alertness and Sedation and the American Society of Anesthesiologists Continuum of Depth of Sedation, at different stages of anesthesia.

**Results:**

Enrollment for this study has been completed and statistical data analyses are currently underway.

**Conclusions:**

The results of this trial will provide information that will simultaneously compare the performance of SNAP II and BIS VISTA devices, with regards to monitoring different anesthesia states among patients.

**ClinicalTrial:**

Clinicaltrials.gov NCT00829803; https://clinicaltrials.gov/ct2/show/NCT00829803 (Archived by WebCite at http://www.webcitation.org/6nmyi8YKO)

## Introduction

Anesthesiologists have relied on patients’ somatic signs (motor response, alteration in respiratory patterns) and autonomic signs (hypertension, tachycardia, lacrimation, sweating), and eliciting responses using tactile and verbal stimulation when assessing patients’ depth of anesthesia and comfort level [1,2]. Based on this data, anesthetic agents are used to achieve hypnotic effects, blocking somatic motor response and suppressing autonomic response to noxious stimulation [1,2]. Although objective physiological signs are easy to observe in nonresponsive patients, these signs may be nonspecific [1].

Intraoperative awareness has been a concern among surgical patients undergoing anesthesia and it may be responsible for postoperative major depression and/or suicide secondary to posttraumatic stress disorder [3,4]. The incidence of intraoperative awareness is approximately 0.1-0.2% among the general surgical population, and approximately 1% among patients with reduced cardiac reserve, or those undergoing cardiac surgery or caesarean section [3-5].

Commercially available electrical monitors have been developed to enhance the assessment of patients’ levels of consciousness, using both low- and high-frequency electroencephalogram (EEG) signals [6,7]. SNAP II is a single-channel EEG device that monitors brain activity [8], and is intended to monitor the state of the brain by acquiring EEG signals [9]. A derived measure provided by the SNAP II index indicates the patient’s brain activity level [9]. The SNAP II index is based on a unique combination of low-frequency (0.1-40 hertz) and high-frequency (80-420 hertz) EEG analysis that is processed for display in a time-based trend [8].

Conversely, the bispectral index (BIS) VISTA is a dual-channel EEG device that monitors brain activity [8]. The BIS VISTA index is based on bispectral analysis of the EEG [8]. BIS VISTA is a dimensionless number between 0 and 100 [8,10] (Table 1). The 40-60 range is suitable for surgical anesthesia, 0 indicates flat line EEG, and 100 indicates that the patient is fully awake [3,10-15]. SNAP II records a value every second, whereas BIS VISTA is usually set up to provide indices every 5 seconds [10,16]. Studies have shown that monitoring a patient’s consciousness level during a procedure leads to a reduction in drug consumption, which helps in earlier recovery and reduces anesthesia-related adverse events [3,15,17].

This clinical study will provide information by simultaneously comparing the performance of SNAP II and BIS VISTA, by assessing their efficacy in monitoring different anesthetic states in patients undergoing general anesthesia.

**Table 1:** BIS VISTA index ranges [10].

Index	BIS VISTA
100	Awake: responds to normal voice
80	Light/moderate sedation: may respond to loud command or mild prodding/shaking
60	General anesthesia: low probability of explicit recall and unresponsive to verbal stimulus
40	Deep hypnotic state
20	Burst suppression
0	Flat line electroencephalogram

## Methods

This is a multicenter, prospective, double-arm, parallel assignment, single-blind study involving patients undergoing elective surgery that requires general anesthesia. The study will examine the predictive value of Stryker Instruments’ SNAP II versus the BIS VISTA indices at various anesthetic states during a surgical procedure under general anesthesia.

Information obtained during the study will be used to identify and evaluate the benefit of using SNAP II in different types of surgical procedures. This data will include electronic records from the devices undergoing study, electronic records from physiological monitor(s), the anesthesia provider’s notes, case notes from a clinical observer, patient demographic information (gender, age, weight, height, medical history, and handedness), and record of Post-Anesthesia Care Unit (PACU) follow-up.

The primary objective of this study is to establish the range of index values for the SNAP II corresponding to each anesthetic state (preinduction, loss of response, maintenance, first purposeful response, and extubation). The secondary objectives will assess the range of index values for BIS VISTA corresponding to each anesthetic state compared to published BIS VISTA range information, and estimate the area under the curve (AUC), sensitivity, and specificity for both devices.

After Institutional Review Board (IRB) approval, all clinical investigation sites will enroll a total of 40 patients. All surgeries will be performed at the following sites: The Ohio State University Medical Center (Columbus, OH); Northwestern University Prentice Women's Hospital (Chicago, IL); and University of Miami Jackson Memorial Hospital (Miami, FL).

Eligibility requirements include: patients 18 to 65 years of age, American Society of Anesthesiologists (ASA) physical status classification system I-III, body mass index <40, weight >41 kilograms, undergoing elective surgery (expected length <4 hours) under general anesthesia, and signing informed consent. Exclusion criteria include: prisoners, pregnant women, taking psychoactive medication within the past 7 days, known alcohol or narcotic abusers within the last 6 months or reporting narcotic use 24 hours prior to surgery, history of any adverse incident with anesthesia, head or neck surgery, recent trauma, active seizure disorder, dementia, Alzheimer’s disease, or active infection.

Data collection for all devices in the study (SNAP II, BIS VISTA, and the physiological monitor) will be performed electronically, enabling the monitors to record case events. Case event recording will be done using a master clock (operating room wall clock) synchronized with the SNAP II device, the BIS VISTA device, the physiological monitor, or a laptop computer used to collect data from the physiological monitor.

Clinical consciousness assessments will be standardized using the Modified Observer’s Assessment of Alertness and Sedation (MOAAS; Table 2 [18]) and the ASA Continuum of Depth of Sedation (Table 3 [19]) criteria.

**Table 2:** Modified Observer’s Assessment of Alertness and Sedation Scale.

Score	Responsiveness
5	Responds readily to name spoken in normal tone
4	Lethargic response to name spoken in normal tone
3	Responds only after name is called loudly and/or repeatedly
2	Responds only after mild prodding or shaking
1	Responds only after painful trapezius squeeze
0	No response after painful trapezius squeeze

**Table 3:** American Society of Anesthesiologists Continuum of Depth of Sedation.

	Minimal Sedation (Anxiolysis)	Moderate Sedation/ Analgesia (Conscious Sedation)	Deep Sedation/ Analgesia	General Anesthesia
Responsiveness	Normal response to verbal stimulation	Purposeful response to verbal or tactile stimulation	Purposeful response following repeated or painful stimulation	Unarousable even with painful stimulus
Airway	Unaffected	No intervention required	Intervention may be required	Intervention often required
Spontaneous ventilation	Unaffected	Adequate	May be inadequate	Frequently inadequate
Cardiovascular function	Unaffected	Usually maintained	Usually maintained	May be impaired

Five anesthetic states will be monitored in this study, as follows:

1. Preinduction baseline: time between application of the electrodes and induction.

2. Loss of response: occurs within the induction phase of anesthesia, defined as the period of time starting with the delivery of the induction agents and ending at the beginning of maintenance. During anesthesia induction, patients will be instructed to hold onto a weighted object as long as possible using a supinated hand. The moment that the object is dropped will be considered the *loss of response* time.

3. Anesthesia maintenance: this time starts 10 minutes after completion of induction medication administration, and ends when the anesthetic agent delivery stops.

4. First purposeful response: defined as a definitive response after anesthesia is discontinued and the MOAAS score is >1 and ASA status is *deep sedation* or lighter.

5. Extubation or laryngeal mask airway (LMA) removal: as per meeting clinical criteria for extubation or discontinuation of the LMA.

Delivery of anesthesia will be standardized, and the use of anesthetic agents will be controlled and carefully documented during the study to avoid confounding results. Tapering of inhalation agents in anticipation of the end of a surgical case will not be allowed, as this could skew study results. Allowable agents for this study are outlined in Textbox 1.

**Agents eligible to be used in the study.**

- Preinduction (anxiolytics) agents such as Versed (0.04 milligrams/kilogram, maximum 2 milligrams).
- Induction agents such as propofol (2 milligrams/kilogram) delivered as a continuous infusion at the rate of 300 micrograms/kilogram/minute; succinylcholine is optional for endotracheal tube placement only.
- Prior to intubation or LMA placement, use up to 0.5 micrograms/kilogram fentanyl, plus timely delivery of succinylcholine if needed.
- After the administration of fentanyl, face mask ventilation (FMV) will be performed with sevoflurane (1.0-1.5 minimum alveolar concentration adjusted per age) for 3 minutes. Partial pressure of carbon dioxide <50 mmHg and minimum peripheral capillary oxygen saturation of 94% will be maintained. Nitrous oxide is not allowed.
- After 3 minutes of FMV, intubation or LMA placement will be performed.
- Optional agents will be used as per investigator criteria and ondansetron (4 milligrams intravenous) and/or other antiemetic will be administered for postoperative nausea and vomiting prophylaxis. Fentanyl will be used as a narcotic only (not as induction or maintenance agent) for intraoperative analgesia. Analgesia upon PACU arrival will follow institutional standard operational procedures (may use drugs other than fentanyl) or toradol (30 milligrams intravenous) will be used as an alternative.
- The following agents/techniques are not allowed during the study: ketamine, total intravenous anesthesia, and prolonged use of neuromuscular blocking agents beyond dose required for intubation or LMA placement.

### Electronic and Clinical Consciousness Level Monitoring

Placement of SNAP II and BIS VISTA electrode arrays will be based on the manufacturers’ recommended locations (as instructed on electrode packaging materials) and never on the same side of the forehead. Placement of the electrode arrays (left or right) will be allocated based on the study patient number indicated by the study design: even patient numbers will have the SNAP II electrode placed on the right side and the BIS VISTA electrode on the left, and odd patient numbers will have the SNAP II electrode placed on the left side and the BIS VISTA electrode on the right. SNAP II electrodes will be placed as follows: circle number 1 (or number 3) at the center of the forehead above the nose approximately 1.5 inches (approximately 4 centimeters) above the bridge of the nose; circle number 2 (middle sensor) over the arch of the eyebrow; and circle number 3 (or number 1) at the end of the eyebrow line just above the temple. BIS VISTA electrodes will be placed as follows: circle number 1 at the center of the forehead, approximately 2 inches (approximately 5 centimeters) above the nose; circle number 4 directly above and parallel to the eyebrow; and circle number 3 on the temple area between corner of eye and hairline.

Preoperative baseline indices from SNAP II and BIS VISTA monitors will be collected to assess the baseline indices and sensors’ impedance checks. Prior to the baseline indices, an impedance check for both devices will be performed and manually recorded. The SNAP II electrode impedances will be checked with a third-party impedance meter to assess the stability of the indices (<10% variation). These preoperative readings will be obtained in the preoperative holding area or in the operating room ten minutes after placing the electrodes from both devices, and before the administration of midazolam; the SNAP II and BIS VISTA baseline index readings will be collected and manually recorded 4 times in a 15 second interval. Consequently, baseline MOAAS and ASA assessments will be performed.

Upon the patient’s arrival in the operating room, electrode impedances from both devices will be checked and recorded again to assess the stability of the indices. Preinduction MOAAS and ASA assessments will be performed, then both electrodes will be connected to their respective monitors before induction time, to assess the indices from both devices 4 times in a 15 second interval.

After the patient shows a loss of response, 4 MOAAS and ASA assessments will be performed at 15 second intervals to confirm the loss of response event. In addition, a manual impedance check of both devices will be performed, and time and value will be recorded to confirm the stability of the indices.

After intubation or LMA placement, MOAAS and ASA assessments will be performed every 2 minutes until sevoflurane administration is discontinued. Furthermore, the time and description of all significant events or maneuvers/procedures performed during maintenance will be recorded.

**Figure 1:**
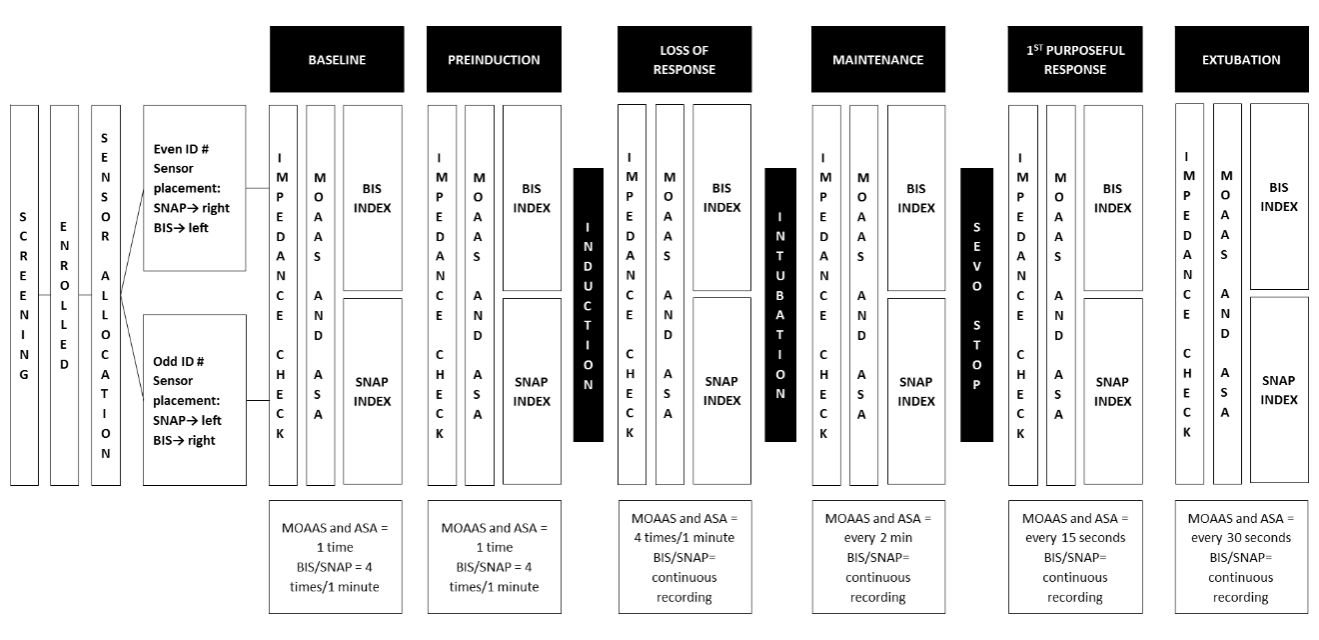
Procedural flow chart. BIS: bispectral index; ASA: American Society of Anesthesiologists Continuum of Depth of Sedation; MOAAS: Modified Observer’s Assessment of Alertness and Sedation; SEVO: Sevoflurane.

Prior to sevoflurane discontinuation, additional impedance checks will be performed on both devices, and the corresponding times and values will be recorded. At incision closure, sevoflurane administration will be discontinued (tapering of sevoflurane will not be allowed). Consequently, MOAAS and ASA assessments will be performed every 15 seconds until the first purposeful response is observed. From this moment, these assessments will be performed every 30 seconds until patients meet clinical criteria for extubation or LMA removal. Additionally, impedance checks will be performed and recorded immediately before extubation or LMA removal time. The study procedure is outlined in Figure 1.

Postsurgical pain assessment will be conducted upon PACU arrival by using a numeric pain rating scale, and asking the patient to rate their pain ranging from 0 to 10: 0 indicates no pain and 10 indicates the worst possible pain. PACU discharge will be determined by institutional standard of practice.

There is no patient follow-up in this study beyond pain assessment in the PACU. Any deviations from the protocol will be documented and the appropriate report will be submitted to the participating IRB and sponsor. All postoperative complications/concurrent medical events and follow-up on existing complication/adverse events will be documented and reported accordingly. The investigator will immediately notify the monitor and the sponsor regarding all adverse product-related events until the patient’s admission to PACU. The event is to be appropriately documented by the investigator on a serious adverse event form.

### Statistical Methods

In this study, for a given pair of clinical states, our goal is to choose a threshold in SNAP II values that best distinguishes the two states, and to precisely estimate the accuracy associated with this threshold. We define this accuracy as the average of the sensitivity and specificity (if an equal number of values for both states exist, this is simply the proportion of the index values during both states correctly classified). This accuracy calculation will first be done for each patient, and then the patient-level accuracies will be averaged to determine an overall estimate of accuracy. We assume an overdispersed binomial variance, which depends not only on the true accuracy but also on a within-patient correlation of the correct classification indicator variables. We will calculate the standard deviation of the overall estimated accuracy under varying values of number of patients, number of SNAP II index values per patient per state, true mean accuracy, and within-patient correlation. Assuming the true accuracy is 0.95, the number of index values per state is at least 10, and the within-patient correlation is 0.25, a sample of 30 patients will result in a standard deviation of approximately 0.02, which we deem sufficiently small.

There are four pairs of clinical states that we wish to distinguish using the SNAP II index values. For each pair of states, the nonparametric estimate of the area under the receiving operating characteristic (ROC) curve will be computed at the patient-level. An overall AUC will be computed as the average of the patient-level values. Confidence intervals (95%) will be computed as mean (standard error 1.96), where the standard error of the mean is based on patient-level values. An overall ROC curve for each pair of states will be estimated by pooling observations across patients. Ranges of typical index values for each of the 5 clinical states will be identified and reported. Correlation between MOAAS values and the SNAP II and BIS VISTA index values will be assessed with the nonparametric Spearman’s rank-based correlation. Only the index value closest in time to each MOAAS value will be used in estimating this correlation. In addition, box plots of SNAP II and BIS VISTA index values versus MOAAS values will be created. Plots showing SNAP II and BIS VISTA index values over time for each patient, with annotated clinical states, will also be created.

Both BIS VISTA and SNAP II index values will be assigned to a clinical state based on the time of index value collection. Only values of SNAP II and BIS VISTA taken at the same time will be used. SNAP II records a value every second, whereas BIS VISTA records a value every 5 seconds, so every fifth SNAP II value will be used in analyses [10,16]. For baseline, loss of response, maintenance, first purposeful response, and extubation, the 10 values of BIS VISTA immediately following the clinical event (covering approximately 50 seconds of time) and the 10 matching SNAP II values will be assigned to that state. All BIS VISTA values and matching SNAP II values obtained from 10 minutes after loss of consciousness until the inhalation anesthesia is turned off will be assigned to maintenance state.

## Results

Enrollment for this study has been completed and statistical data analyses are currently underway.

## Discussion

This part of the study will be elaborated when data analyses are completed. The results of this clinical trial will provide information by simultaneously comparing the performance of SNAP II and BIS VISTA by assessing their efficacy in monitoring different anesthetic states in patients undergoing general anesthesia. Due to the complexity of the study procedures and anesthesia regimen used for this trial, we anticipate a moderate to high incidence of screen failures during enrolment.

## Abbreviations

ASA: American Society of Anesthesiologists
AUC: area under the curve
BIS: bispectral index
EEG: electroencephalogram
FMV: face mask ventilation
IRB: Institutional Review Board
LMA: laryngeal mask airway
MOAAS: Modified Observer’s Assessment of Alertness and Sedation
PACU: Post-Anesthesia Care Unit
ROC: receiving operating characteristic
